# Titanium Oxide: A Bioactive Factor in Osteoblast Differentiation

**DOI:** 10.1155/2015/357653

**Published:** 2015-11-18

**Authors:** P. Santiago-Medina, P. A. Sundaram, N. Diffoot-Carlo

**Affiliations:** ^1^Department of Biology, University of Puerto Rico, Mayaguez, PR 00680, USA; ^2^Department of Mechanical Engineering, University of Puerto Rico, Mayaguez, PR 00680, USA

## Abstract

Titanium and titanium alloys are currently accepted as the gold standard in dental applications. Their excellent biocompatibility has been attributed to the inert titanium surface through the formation of a thin native oxide which has been correlated to the excellent corrosion resistance of this material in body fluids. Whether this titanium oxide layer is essential to the outstanding biocompatibility of titanium surfaces in orthopedic biomaterial applications is still a moot point. To study this critical aspect further, human fetal osteoblasts were cultured on thermally oxidized and microarc oxidized (MAO) surfaces and cell differentiation, a key indicator in bone tissue growth, was quantified by measuring the expression of alkaline phosphatase (ALP) using a commercial assay kit. Cell attachment was similar on all the oxidized surfaces although ALP expression was highest on the oxidized titanium alloy surfaces. Untreated titanium alloy surfaces showed a distinctly lower degree of ALP activity. This indicates that titanium oxide clearly upregulates ALP expression in human fetal osteoblasts and may be a key bioactive factor that causes the excellent biocompatibility of titanium alloys. This result may make it imperative to incorporate titanium oxide in all hard tissue applications involving titanium and other alloys.

## 1. Introduction

Titanium alloys have become the most popular metallic biomaterials in dental applications because of their excellent biocompatibility [1]. This is attributed to the inert nature of the titanium surface due to the formation of a thin native titanium oxide layer [2] which also provides excellent corrosion resistance. Although titanium alloys have virtually replaced other metallic biomaterials in dental implant applications, currently there is little insight into the reasons for this excellent biocompatibility of titanium surfaces. A number of studies have pointed out various factors that contribute to the biocompatibility of titanium or of modified titanium surfaces [3–6]. While these studies have investigated both proliferation and differentiation of osteoblast cell lines evidenced in many instances by gene expression to corroborate biocompatibility, the emphasis has been on physical factors such as roughness, texture, wettability, or substrate microstructural features. Some importance has been paid to the substrate composition, whether Ti or Ti alloy, and the makeup of the surface modified layer. In recent work [3], the plausible role of titanium oxide in contributing to this outstanding biocompatibility was observed. In studying titanium alloys, it was noticed that ALP activity was higher on oxidized titanium alloys compared to corresponding untreated materials [7, 8]. Despite having a good understanding of the signaling pathways in osteoblast differentiation [4], the effect of titanium oxide on osteoblast differentiation has not been fully researched even though the fact that a thin native titanium oxide layer forms on all titanium alloys is well known and a large amount of research has been conducted on these popular biomaterials.

It is still unclear at the present time whether osteoblast differentiation is affected by titanium oxide or by the oxygen released from the titanium oxide. A recent report suggests that oxygen tension, in and of itself, has a strong effect on osteoblast differentiation and may in fact regulate this process [5], and hypoxic cell cultures demonstrated a lower level of mineralization resulting in a more chondrogenic tissue in comparison to higher levels of oxygen in cell cultures [6]. Healing of fractures has also been reported much earlier to be delayed in the absence of oxygen [9]. In contrast, reactive oxygen species (ROS) has been reported to suppress bone formation and stimulate bone resorption [10]. Osteoblast differentiation involves a complex molecular pathway consisting of various transcription factors and it is well known that many transitional stages comprise the pathway for this process and that several signaling molecules play key roles in overall skeletal development [4]. It is currently unknown if titanium oxide plays a critical role in any step of the signaling cascade. Understanding the manner in which titanium oxide affects osteoblast differentiation may be critical in titanium implantology in terms of reducing hospitalization time and formulating efficient therapeutic procedures.

In this study, hFOB cells were cultured for 10 days on the oxide formed on the surface of two titanium-based alloys from two different methods of oxidation and the degree of osteoblast differentiation was measured through quantification of ALP activity using a commercial assay kit to compare with unoxidized titanium alloy surfaces in an effort to determine if titanium oxide indeed played a role in the differentiation process.

## 2. Materials and Methods

### 2.1. Preparation of Titanium Disks

Various samples of the two titanium-based alloys, gamma-TiAl (*γ*-TiAl [Ti-48Al-2Cr-2Nb (at.%)]) and Ti-6Al-4V (wt.%), were machined in the form of 7 mm diameter cylindrical rods. From these, disks with an approximate thickness of 1 mm were obtained using a slow-speed diamond saw (Buehler). Both surfaces of the disks were ground using 240, 320, 600, and 1200 grit silicon carbide paper in an Ecomet 3 (Buehler). These metal disks were then sonicated in 0.8% Alconox (Fisher, Pittsburgh, Pennsylvania) and 70% ethanol for 10 minutes each, rinsed with deionized water, and dried with a hot-air blow-dryer.

### 2.2. Thermal Oxidation

Both *γ*-TiAl and Ti-6Al-4V disks were oxidized in a laboratory furnace (CM Furnaces Inc.) in air at 500°C or 800°C for 1 h and later placed in 48-well cell culture plates (Corning). The nomenclature followed in this paper is as follows: *γ*-TiAl and Ti-6Al-4V disks oxidized at 500°C and 800°C are hereafter referred to as GTi5, GTi8, TiV5, and TiV8, respectively, while untreated disks are designated as GTi and TiV. Atomic force microscopy (AFM) was used to obtain average surface roughness values of the oxidized surfaces. These are given in Table 1.

### 2.3. Micro Arc Oxidation

Other *γ*-TiAl and Ti-6Al-4V disk samples were processed using micro arc oxidation (MAO). For the MAO process, a stainless steel beaker was used as the cathode, while the titanium disk (either *γ*-TiAl or Ti-6Al-4V) was used as the anode. Each sample was mounted in a titanium holder specially designed to allow complete exposure of the sample to the electrolyte [11]. A* Hoefer PS300-B* high voltage power supply (300 V; 500 mA) was operated in galvanostatic mode in order to form the titanium oxide film on the sample surface using the MAO process. Process conditions of sample current of 200 mA and 225 mA for durations of 3 and 4 minutes for each case were utilized based on an earlier study [11]. After treatment, the samples were rinsed with distilled water and then dried with a blow dryer. The micro arc oxidized samples will be henceforth referred to as MAOGTi for the *γ*-TiAl samples and the MAOTiV for the Ti-6Al-4V samples, respectively. The oxides formed on both *γ*-TiAl and Ti-6Al-4V as a result of the MAO treatment for the applied process conditions are mainly rutile and anatase as reported earlier [11, 12]. For thermal oxidation, alumina is the dominant oxide formed on *γ*-TiAl at 500°C, while rutile is dominant at 800°C [13–16]. For Ti-6Al-4V, thermal oxidation at 500°C produces a combination of an oxide diffused Ti(O) and rutile where the latter phase appears to grow at high temperatures between 650°C and 800°C [17]. The average roughness values of these surfaces were extracted from topography analysis using AFM and presented in Table 1.

### 2.4. Human Fetal Osteoblast Cell Line

Human osteoblast cells, cell line hFOB 1.19 (ATCC, Manassas, Virginia), were cultured in 90% Dulbecco's Modified Eagle's Medium Nutrient Mixture F-12 Ham (DMEM) (Sigma-Aldrich, St. Louis, Missouri) with 2.5 mM L-Glutamine and 15 mM Hepes, without phenol red, supplemented with 0.3 mg/mL G-418 (Calbiochem, San Diego, California) and 10% Fetal Bovine Serum (FBS) (Hyclone, Logan, Utah). Cells were grown in 25 cm^2^ plastic culture flasks (Corning, Corning, New York) and incubated at 33.5°C until confluence. At approximately 100% confluence, cells were washed three times with Phosphate Buffer Saline (PBS) solution (137 mM NaCl, 2.7 mM KCl, 4.3 mM Na_2_HPO_4_, and 1.4 mM KH_2_HPO_4_) and harvested using 0.25% trypsin-0.53 mM EDTA (Gibco, Gaithersburg, Maryland) at 37°C for 5 min. Cells were then pelleted by low speed centrifugation (3,300 rpm) for 5 minutes and subcultured at a 1 : 3 ratio.

### 2.5. Alkaline Phosphatase Assay

Cells were seeded in 48-well plates (Becton, Dickinson, Lincoln Park, NJ) at a density of 5 × 10^4^ cells/cm^2^ on TiV, TiV5, TiV8, GTi, GTi5, GTi8, MAOTiV, and MAOGTi disks (7 mm in diameter), using the commercial Alkaline Phosphatase Colorimetric Assay Kit (ab83369, Abcam) in order to evaluate osteoblast differentiation quantitatively on thermally oxidized, micro arc oxidized, and untreated *γ*-TiAl and Ti-6Al-4V disks. Samples were incubated for 3 days at 33.5°C and then for 7 days at 39.5°C to allow osteoblast differentiation. hFOB cells grown on coverslips were used as controls. Modifications to the suggested protocol were made to achieve a more efficient cell lysis, including washing the samples carefully three times with PBS and homogenizing in 60 *μ*L of the Assay Buffer. Also, Triton X-100 (80 *μ*L) was utilized to lyse the cells for an efficient measurement of intracellular ALP. Stop solution (20 *μ*L) was added to terminate ALP activity in the sample. The solution in each well was transferred to a 96-well plate (Becton, Dickinson, Lincoln Park, NJ).* p*NPP solution (50 *μ*L) was added to each well containing the test samples and background controls. The reaction was incubated for 60 minutes at 25°C, protected from light.

A standard curve was generated to determine the concentration of ALP activity in the sample for which 40 *μ*L of the 5 mM* p*NPP solution was diluted with 160 *μ*L Assay Buffer to generate a 1 mM* p*NPP standard. 0, 4, 8, 12, 16, and 20 *μ*L were added to 96-well plate in duplicate to generate 0, 4, 8, 12, 16, and 20 nmol/well* p*NPP standard. The final volume was brought to 120 *μ*L with Assay Buffer. ALP enzyme solution (10 *μ*L) was added to each well containing the* p*NPP standard. The reaction was incubated for 60 minutes at 25°C, protected from light. All reactions were stopped by adding 20 *μ*L Stop Solution to each standard and sample reaction except the sample background control reaction (since 20 *μ*L Stop Solution had been added to the background control when prepared previously). The optical density was measured at 405 nm in a microplate reader. The background was corrected by subtracting the value derived from the zero (0) standards from all standards, samples, and sample background control. The* p*NPP standard curve was plotted and the sample readings were applied to the standard curve to get the amount of* p*NPP generated. ALP activity of the test samples was calculated using the equation, ALP activity (U/mL) = *A*/*V*/*T*, where *A* is amount of* p*NPP generated by samples (in *μ*mol), *V* is volume of sample added to the assay well (in mL), and *T* is reaction time (in minutes).

### 2.6. Statistical Analysis

Three independent experiments were performed for each ALP assay, and since each experiment had three replicates, a total of nine replicates per surface were evaluated (MAO *γ*-TiAl, thermally oxidized *γ*-TiAl, untreated *γ*-TiAl, MAO Ti-6Al-4V, thermally oxidized Ti-6Al-4V, untreated Ti-6Al-4V, and control glass coverslips) for a 10-day period of culture. The data from the ALP assay is presented as the mean ± standard deviation (SD) of the optical density of differentiated cells on the different surfaces corresponding to the amount of alkaline phosphatase detected. Each value represents the mean of three measurements of cell differentiation performed on a specific surface as mentioned above. A factorial analysis of variance (ANOVA) was used to assess the significant interactions between type of metal (*γ*-TiAl or Ti-6Al-4V) and type of surface treatment (micro arc oxidization at 200 mA, 3 min, 200 mA, 4 min, 225 mA, 3 min, and 225 mA, 4 min; thermal oxidization at 500°C and 800°C). All significant interactions were graphically analyzed and, in addition, a randomized block design was performed to reduce the variance in the data. Furthermore, a contrast test was performed to compare the type of metal (*γ*-TiAl and Ti-6Al-4V) with the surface treatments. Significant differences in cell differentiation on the type of metal and surfaces tested were confirmed using the LSD Fisher test. All analyses were performed using Infostat (Infostat Inc.).* p* values < 0.05 were considered to be statistically significant.

## 3. Results

**Figure 1:**
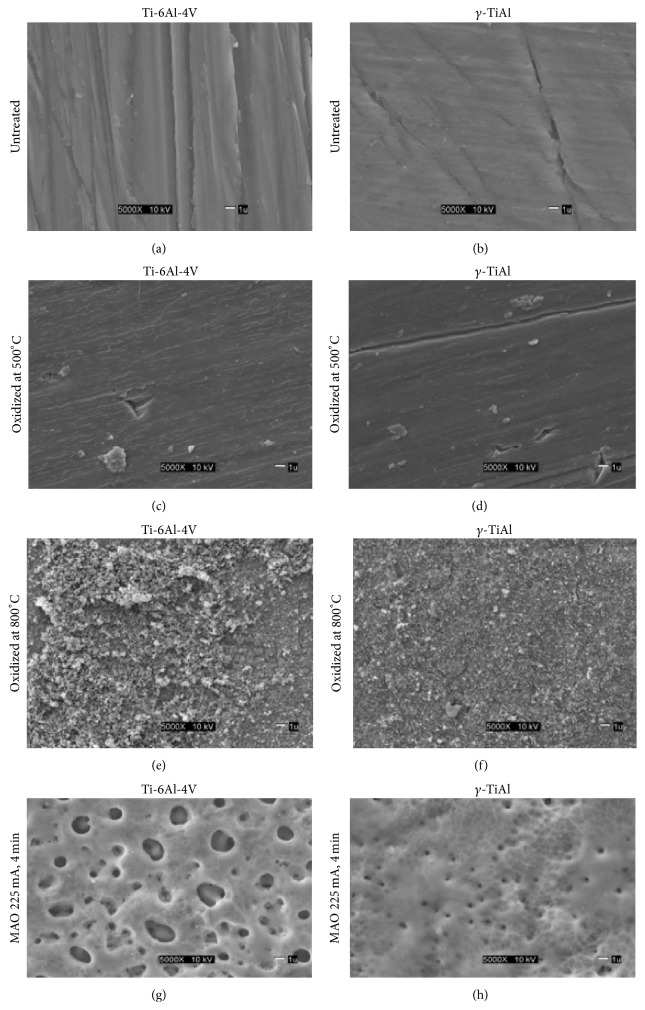
SEM images of Ti-6Al-4V and *γ*-TiAl alloys. (a), (b): untreated, (c), (d): oxidized at 500°C, (e), (f): oxidized at 800°C, and (g), (h): MAO at 225 mA, 4 mins.

SEM images of glass coverslips, untreated Ti-6Al-4V and *γ*-TiAl surfaces (TiV and GTi), micro arc oxidized Ti-6Al-4V and *γ*-TiAl surfaces (MAOTiV and MAOGTi), and thermally oxidized Ti-6Al-4V and *γ*-TiAl surfaces (TiV5, TiV8, GTi5, and GTi8), are shown in Figure 1. GTi and TiV (Figures 1(a) and 1(b)) exhibit a smoother surface of the passive oxide layer formed instantaneously on Ti alloys. In contrast, rounded surface structures were visible in TiV5, suggesting clusters of oxide granules. GTi8 and TiV8, on the other hand, exhibited a rougher surface in comparison to the other samples (Figures 1(e) and 1(f)). Larger oxide granules were present on TiV8 (Figure 1(e)) compared to those on GTi8 (Figure 1(f)), conferring an irregular appearance to this surface and suggesting that TiV8 oxide layer could possibly be thicker. The MAO surfaces for Ti-6Al-4V (MAOTiV) (Figure 1(g)) demonstrated a number of large pores on the oxide surface typical of similar treatments on Ti alloys [18, 19]. Although pores were also clearly visible on the surface of MAOGTi, these were smaller and on the average in the submicron range.

**Figure 2:**
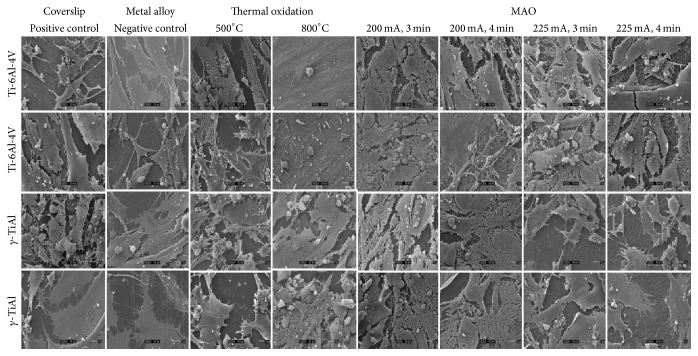
SEM micrographs of hFOB 1.19 cells on a glass coverslip (positive control), GTi and TiV (untreated alloys), thermally oxidized TiV5 and GTi5 (500°C), TiV8 and GTi8 (800°C), micro arc oxidized (MAO) TiV (200 mA and 225 mA at 3 min and 4 min), and MAOGTi (200 mA and 225 mA at 3 min and 4 min) disks. Magnification 1000x.

**Figure 3:**
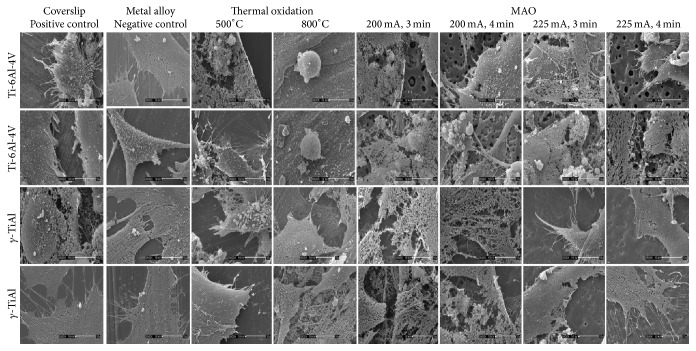
SEM micrographs of hFOB 1.19 cells seeded on a glass coverslip (positive control), GTi and TiV (untreated alloys), TiV5 and GTi5 (500°C), TiV8 and GTi8 (800°C), MAOTiV (200 mA and 225 mA at 3 min and 4 min), and MAOGTi (200 mA and 225 mA at 3 min and 4 min) disks and incubated for 10 days (3 days at 33.5°C and subsequently 7 days at 39.5°C). Magnification 3500x.

SEM images shown in Figure 2 indicate that hFOB 1.19 cells grew normally on the surfaces of untreated *γ*-TiAl and Ti-6Al-4V disks. Cell attachment and proliferation were similar on both metal surfaces, suggesting normal growth, cell confluence, and attachment under* in vitro* conditions. The osteoblast cells were spread and flattened on the glass coverslips exhibiting such close contact with each other, that detection of cellular boundaries was difficult. Fibrous networks corresponding to fibrillar collagen, the main component of bone ECM, which aid in the adhesion of cells and important for the proper assembly of the extracellular matrix, are visible lending further testimony to the normal growth of osteoblasts on these surfaces. The ECM, which serves as a calcium phosphate reservoir, provides support for the cells, offers protection, and is very important in homeostasis, appears to be forming copiously [20]. Included are nodules of mineralization with a sponge-like morphology and intimately associated with the fibrillar network and scattered throughout the samples [21]. The maturation of the ECM is evidenced by the presence of fibrous networks associated with cells and an increase in nodules of mineralization. On the thermally oxidized Ti-6Al-4V and *γ*-TiAl alloys at 500°C, cellular attachment and proliferation were similarly observed. A cell multilayer, constituted by elongated and polygonal cells with some round shaped cells (Figure 2), along with the presence of a few small rounded structures which may correspond to mineral nodules, was observed. At higher magnification (Figure 3), the mineral nodules appeared to be in close contact with cells and had a sponge-like appearance. On the thermally oxidized Ti-6Al-4V alloys at 800°C, only irregular structures were observed. There was cell debris indicative of cytotoxicity of the oxide (Figure 3) in agreement with an earlier report [20]. However, on thermally oxidized *γ*-TiAl alloys, fibrous networks and mineralized nodules were observed on elongated cells (see Figure 3). Slender cytoplasmic projections (filopodia) extended from the cells in all directions on all micro arc oxidized Ti-6Al-4V and *γ*-TiAl disks, confirming the biocompatibility of the substrate materials [12]. In addition, sheet-like cytoplasmic protrusions extending from the cell body in all directions suggest the ability of cellular movement, spreading of the cells on the substrate, and/or the fact that cellular division (mitosis) may be occurring. The presence of mineralized nodules again suggests the maturation of the ECM and the formation of bone-like tissue indicating osteoblast differentiation evidenced by a high degree of ALP activity. Taken together, normal cell attachment and proliferation on all the surfaces with the exception of TiV8 indicate the excellent biocompatibility of control, untreated, and treated titanium alloy surfaces.

**Figure 4:**
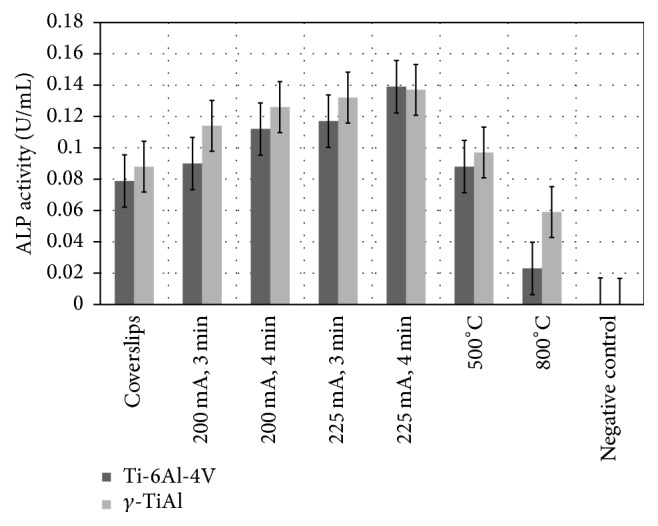
Alkaline phosphatase activity on thermally oxidized and MAO treated Ti-6Al-4V and *γ*-TiAl alloys. ALP activity on positive control (glass coverslips) and untreated Ti alloys is also shown.

Standard calibration curves were used to extrapolate the values of alkaline phosphatase (ALP) activity on experimental disks 10 days after seeding based on the alkaline phosphatase assay. The ALP activity, measured as described in Section 2, is plotted in Figure 4 for the various sample surfaces that were utilized in the experiment to measure osteoblast differentiation. It is clear that little ALP activity is observed on the untreated Ti alloy samples while the positive controls (glass coverslips) indicate reasonable ALP activity corresponding to osteoblast differentiation. For the surface treatments of thermal oxidation and MAO, the Ti samples clearly showed relatively higher ALP activity. The highest ALP activity is observed for the Ti alloys subjected to the MAO treatment. As an exception, it must be noted that the ALP activity on Ti-6Al-4V oxidized at 800°C is rather low compared to the other treated samples. ANOVA revealed significant interactions between the factors tested (type of metal and treatment) and ALP activity. The interaction between the type of metal (*γ*-TiAl or Ti-6Al-4V) and surface treatment (micro arc oxidation at 200 mA, 3 min, 200 mA, 4 min, 225 mA, 3 min, and 225 mA, 4 min; thermal oxidation at 500°C or 800°C) was significant (*p* < 0.05). There were also significant differences in the amount of alkaline phosphatase detected among these six surfaces studied after 10 days of incubation at 33.5°C and 39.5°C, respectively. Additionally, alkaline phosphatase activity was lower on the positive control (glass coverslips) and even much lower on untreated titanium alloy surfaces in comparison with the micro arc and thermally oxidized alloys. Furthermore, ALP activity increased in *γ*-TiAl and Ti-6Al-4V alloys and in the MAO treatments where the samples were exposed for longer process times to current density. Although qualitatively there were no significant differences in the number of osteoblast cells attached on the micro arc oxidized or thermally oxidized surfaces collectively, ALP activity was significantly higher on the cell cultures that grew on the micro arc oxidized surfaces in comparison to the thermally oxidized substrates (see Figure 4). Surface roughness of the oxidized surfaces does not appear to show a correlation with ALP activity.

## 4. Discussion

hFOB adherence is clearly excellent on all surfaces with the exception of TiV8 which may possibly be due to the cellular response to toxic compounds or harmful ions such as vanadium in the oxide layer as a result of thermal oxidation [22]. As such, titanium oxide alone does not pose problems of cytotoxicity since cells did attach and proliferate on surfaces of both alloys subject to oxidation at 500°C and also *γ*-TiAl oxidized at 800°C where the oxide formed is predominantly composed of titanium oxide in the form of rutile or anatase [23]. An earlier study showed normal cell attachment on TiV8 2 days after seeding but cell debris as a result of subsequent cell death for longer periods of incubation [20].

Osteoblast differentiation, on the other hand, occurred to different extents on all the substrates as measured by ALP activity. Osteoblast differentiation is a well-coordinated physiological process occurring in three stages which include cell proliferation, ECM production and maturation, and matrix mineralization [24]. Correspondingly, proliferative osteoprogenitors such as* Msx2* and* RP59* are expressed in the first stage, followed by* Runx2*,* Osx*, and OC (osteocalcin) during the later stages in this process of differentiation. During proliferation, Col 1 and ALP are detected earlier on followed by the secretion of RGD containing proteins such as bone sialoproteins (BSP) and osteopontin (OP) and culminating in the synthesis of OC in the last stage of differentiation. Bone morphogenetic proteins (BMPs) and various members of TGF-*β* family are secreted by the osteoblast cells and, once sequestered in the extracellular matrix (ECM), these have also been reported to be critical for osteoblast differentiation [25]. Interaction between Type I collagen and *α2β1* integrin activates a mitogen-activated protein kinase (MAPK) pathway which results in the phosphorylation and activation of* Cbfa1*, in turn stimulating the differentiation of osteoblasts. The ECM which contains BMPs can then induce ALP activity in preosteoblasts [26]. During osteogenesis, the lack of expression of* Runx2* and* Osx* may result in the formation of demineralized bone, although data suggests that these transcriptional factors act independently of each other [27]. Expression of* Runx2* along with* Cbfβ* and alkaline phosphatase has been found to be critical in the early stage of differentiation while* Osx* becomes very important in the later stage of osteoblast differentiation. While, in the present study, cell attachment and proliferation occur normally on all the substrates, except for TiV8, alkaline phosphatase activity measurements indicate that osteoblast differentiation varies depending on the nature of the substrate. The presence of mineral nodules in the ECM on the titanium sample surfaces provides physical evidence to corroborate the activity of osteoblasts in one of their functional stages after maturation as observed in osteoblast cultures at longer periods of incubation [28]. Ti-6Al-4V and *γ*-TiAl thermally oxidized disks at 500°C showed both irregular and rounded mineralized structures on the surface. Similar cell morphology and function were observed for the MAO surfaces for both alloys, suggesting the ability of these surfaces to also promulgate differentiation.

The fact that ALP activity was significantly lower on the untreated alloys (TiV, GTi) suggests that the titanium oxide formed on the surface of these alloys may be strongly correlated with the differentiation of the osteoblasts. It was demonstrated in an earlier study that the oxide layer on MAO treated alloys consists of titanium oxide with peaks for both anatase and rutile phases in all the coating conditions applied in this study [11]. Both anatase and rutile have been shown to be beneficial in enhancing nucleation and subsequent hydroxyapatite (HA) precipitation, thereby increasing bioactivity of the titanium surface [29]. The results from the present study also suggest that the incorporation of calcium (Ca) and phosphorus (P) into the titanium oxide may be favorable for cell differentiation. While Ca and P are important in the observation of increased ALP activity on MAO treated surfaces, the Ti alloys samples which were thermally oxidized at 500°C (devoid of Ca and P) also show a reasonably high ALP activity compared to control glass coverslips. A recent study has shown higher elemental oxygen concentration and higher water wettability on TiO_2_ surfaces when compared to bare titanium surfaces resulting in a twofold increase in ALP activity and mineralized nodule area [30]. It also appears that the topography of bioengineered titanium surfaces affects gene expression and phenotypic response of osteoprogenitor cells [31]. Higher ALP activity on surfaces containing titanium oxide may possibly be correlated with the surface topography of the substrate which may affect cell proliferation and differentiation [31, 32]. Thus it may be argued that the osteoblast differentiation does not depend solely on Ca and P ions but more so on the presence of titanium oxide. In contrast, another research suggests cell cytotoxicity due to TiO_2_ resulting from the interaction between TiO_2_ nanoparticles and the lysosomal compartment, independently of the known apoptotic signaling pathways [33]. However, this has not been fully studied. In addition, TiO_2_ has been reported to possess antibacterial characteristics in stark contrast to its positive biocompatibility [34].

Although it is clear from this study that titanium oxide increases the ALP activity in osteoblasts, the mechanism associated with this process is still unknown. It was proposed that BMP-2 controls alkaline phosphatase expression and osteoblast mineralization by a Wnt autocrine loop in mesenchymal stem cells (MSCs) [35]. Among the factors that regulate MSC growth and differentiation are soluble factors and cell-substrate interactions, although little is known about the molecular mechanisms by which soluble and substrate signals regulate MSC function. These authors showed that the commitment of human MSCs to the osteogenic and adipogenic lineages* in vitro* involves signaling by mitogen-activated protein kinase (MAPK) pathways. In particular, it was found that dexamethasone, ascorbic acid, and *β*-glycerophosphate induce MSC differentiation by regulating the extracellular signal-regulated kinase (ERK1/2) cascade. Furthermore, blocking the ERK1/2 pathway inhibits osteogenic differentiation of MSCs and leads to adipogenesis. Thus MAPK pathways, which are generally activated by growth factors/cytokines and integrin-mediated cell adhesion, play a critical role in directing MSC commitment. MAPK pathways are also activated by physical stimuli to regulate the function of a variety of cell types, including bone cells. In bone, it has been proposed that mature cells such as osteocytes and osteoblasts are responsible for sensing and responding to mechanical stimuli [36]. It is unknown whether the progenitors that give rise to these cells are responsive to mechanical signals. Various signaling pathways, including BMP, Wnt, and notch, regulate bone homeostasis. It is difficult at the present time to determine which of these is affected by titanium oxide to the extent of upregulating ALP activity. While it is clearly demonstrated that hypoxia suppresses osteoblast differentiation and as a consequence bone formation, the mode by which TiO_2_ increases ALP activity is not clear cut. One may speculate that a chemical reaction between TiO_2_ and the culture medium may somehow result in the release of oxygen and this normoxia has a positive impact on osteoblast differentiation. However, it is clear that TiO_2_ is a bioactive factor which indeed upregulates ALP activity, although the manner in which the oxide indeed participates in the biochemical signaling cascade which occurs during differentiation does require further study. This information may be vital in the future of implantology in accelerating fracture healing and other tissue regenerative processes in dental and orthopedic applications. If titanium oxide indeed upregulates osteoblast differentiation, the manufacturers of titanium-based implants would find it advantageous to incorporate titanium oxide as a coating on every surface layer in contact with the tissue side of the implant. Clearly, further research is needed to interrogate the empirical ability of titanium oxide to preferentially favor osteoblast differentiation or at least ALP activity.

## 5. Conclusions

1. ALP activity is much higher on oxidized surfaces of both titanium alloys compared to untreated surfaces most probably due to the presence of titanium oxide.
2. The higher ALP activity on micro arc oxidized surfaces is attributed to the Ca and P content which are not present in the thermally oxidized titanium alloys.
3. The mechanism for the upregulation of ALP due to titanium oxide needs further study although it is clear that TiO_2_ is a bioactive factor in osteoblast differentiation.

## Figures and Tables

**Table 1:** Average roughness values (in *μ*m) measured on Ti alloy sample surfaces for various treatments using AFM.

Treatment	Ti-6Al-4V	*γ*-TiAl
None	51.74	49.30
Oxidation at 500°C	102.40	31.34
Oxidation at 800°C	318.80	65.88
MAO 200 mA, 3 min	246.30	174.50
MAO 200 mA, 4 min	247.60	185.90
MAO 225 mA, 3 min	213.30	137.50
MAO 225 mA, 4 min	301.70	189.80
